# Healing of bronchopleural fistula using a modified Dumon stent: a case report

**DOI:** 10.1186/1749-8090-1-16

**Published:** 2006-06-23

**Authors:** Giorgio Maria Ferraroli, Alberto Testori, Ugo Cioffi, Matilde De Simone, Marco Alloisio, Maurizio Galliera, Michele M Ciulla, Gianni Ravasi

**Affiliations:** 1 Department of Thoracic Surgery – Istituto Clinico Humanitas – Rozzano (MI) –, Italy; 2 Department of Surgery, Fondazione IRCCS, Ospedale Maggiore Policlinico, Maggiagalli e Regina Elena, Milano, Italy; 3 Istituto di Medicina Cardiovascolare, University of Milan, Centro di Fisiologia clinica e Ipertensione, Fondazione IRCCS, Ospedale Maggiore Policlinico, Maggiagalli e Regina Elena, Milano, Italy

## Abstract

**Background:**

Brochopleural fistula following lung resection is a therapeuric challenge for thoracic surgeons.

**Case presentation:**

We describe a case of late bronchopleural fistula after right extrapleural pneumonectomy for malignant mesothelioma. Bronchoscopic attempts to repair it were unsuccessful.

**Conclusion:**

The use of a modified Y Dumon stent associated with glue apposition on the bronchial stump allowed us to close the fistula without the need of any surgical repair.

## Background

The appearance of a bronchopleural fistula is a well-known and frightening complication of pulmonary surgery. Different methods have been used to solve this problem conservatively, from bronchial gluing [1] to stent placement. The latter includes the self-expandable stent [2] or classic Dumon stent [3]. In this case we used a modified Y Dumon stent in an attempt to close a bronchopleural fistula which appeared 10 months after a right extrapleural pneumonectomy for malignant mesothelioma.

## Case presentation

A 64-year old man was referred to our department for right recurrent pleural effusion. A CT scan showed thickened right parietal pleura with a minimal pleural effusion, but no pulmonary nodules or lymphadenopathies or distant metastases. Because of negative cytology malignancy, the patient underwent a thoracoscopic pleural biopsy on December 2004 that showed an epithelioid monophasic pleural mesothelioma, followed by talc pleurodesis at the end of the procedure.

Three weeks later the patient underwent a right extrapleural pneumonectomy with coverage of the bronchial stump with a flap of thymic tissue. The pathological examination confirmed a monophasic epithelioid pleural mesothelioma, with lung and pericardial fat infiltration, but no invasion of the diaphragm or pericardium (pT3 N0 M0). The postoperative period was uneventful.

**Figure 1:**
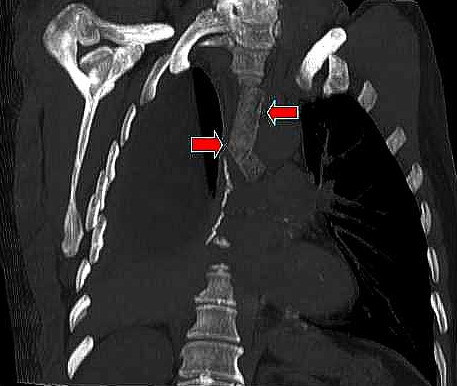
CT multiplanar reformation (MPR), oblique coronal view, with lung window clearly demonstrating patency of the left arm of the of the modified Dumon stent (red arrows).

**Figure 2:**
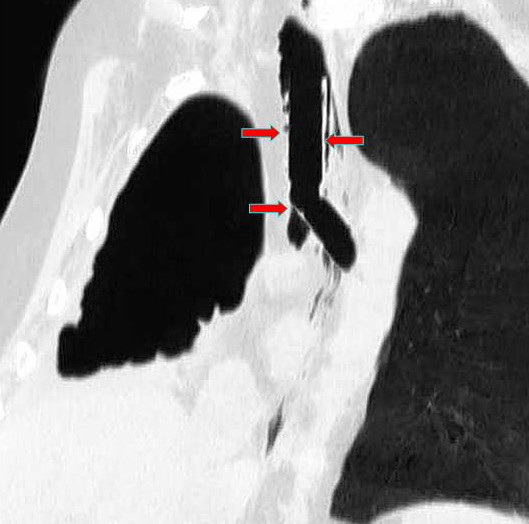
CT multiplanar reformation (MPR), oblique coronal view, after stent placement. The tracheal and bronchial arms of the Dumon stent (red arrows) tightly adhere to the tracheobronchial wall. The covered portion of the stent completely excludes the bronchial stump of about 1 cm length.

Ten months later the patient complained of a fever and cough. The diagnosis of broncho-pleural fistula was made immediately after the first appearance of cough and fever by bronchoscopy that clearly showed a 2-mm fistula on the mediastinal side of the stump confirmed by the methilene blue passage from bronchus to the chest tube. Rapid fluid drainage and antibiotic therapy allowed us to cure the infection of the pleural space avoiding the formation of empyema. After chest tube drainage, the attempt to conservatively close the fistula using biological glue was unsuccessful. So we decided to use a modified Y Dumon stent (Tracheobronxane Y; Novatech SA, La Ciotat Cedex France) to exclude the right stump. The right arm of the Y was shortened and the open end was closed with a silicone material from which the stent was fabricated, and then inserted in the tracheobronchial tree trough a rigid bronchoscope. The tracheal part was 18 mm in width and 4 cm in length, while the left bronchial arm was 16 mm in width and 2 cm in length. Under rigid bronchoscopy, we first introduced the biological glue (CoSeal, Baxter Healthcare Corporation, Fremont, CA – USA) all over the bronchial stump that was of 1 cm lenght, followed by the insertion of the modified Y Dumon stent (Fig. 1–2).

The negative cultures from the pleural fluid allowed us to remove the chest drain and to discharge the patient on the 3rd postoperative day. The patient was uneventful (no empyema or pneumonitis verified) until he died 4 months later for pulmonary emboli. The post-mortem examination showed a massive pulmonary thrombus in the left main pulmonary artery which explain the sudden respiratory insufficiency and death at home and an initial relapse of mesothelioma in the posterior part of chest wall, without signs of infections.

## Conclusion

Biologic glue has been used to treat bronchopleural fistulas, and its efficacy has been demonstrated [1]. When no results are obtained with the biological glue, the Dumon stent is an acceptable option to exclude the bronchial stump from ventilation and to prevent the fistula from staying open, because of the positive pressure exerted on it during breathing, as well as to avoid aspiration pneumonia and the maintenance of empyema [3].

Usually, it is more difficult to treat a fistula on a main bronchus, both for the presence of a single lung and the challenge of inserting the appropriate stent for the changed anatomy following a pneumonectomy. A self-expandable covered tracheobronchial stent could solve this problem for its ability to adequately fit the tracheal and bronchial size as well described in a previous report [2].

The use of a modified Y Dumon stent has some other advantages. First, it can be removed when the patient does not tolerate a foreign body in the bronchial tree; second, it does not give frightening complications such as erosion of the mucosa, with the possibility of perforation and/or severe bleeding.

The only points for careful consideration in this kind of stent are to adequately evaluate the size of the trachea and bronchus, to avoid air or fluid filtration and then the possibility to keep the fistula patent. This has already been well described in other papers where the same kind of modified Dumon stent was used [4,5]. We used the stent as a first choice, closing the fistula immediately after its appearance without any previous surgical procedure as in the case reported by Tsukada and coll. [5].

It is our opinion that this kind of procedure could be useful in the treatment of main bronchus pleural fistulas, and it could be even used in larger ones, eventually associated with apposition of several devices (glue, atoxysclerol, TachoSil, etc.). We also believe that this stent could be performed as a first choice when the fistula appears, thereby avoiding any surgical procedure.

## Competing interests

The author(s) declare that they have no competing interests.

## Authors' contributions

GMF, AT, MG enrolled the patient for clinical and surgical aspects, drafting the article.

UC, MDS. MMC drafting the article, critical revision of the article.

AT, MA, GR performed surgical operations, critical revision of the article, final approval of the version.
